# Evaluation of Hemodynamic Changes and Fluid Response during Anesthesia in Lumbar Disc Hernias with Pleth Variability Index (PVI)

**DOI:** 10.3390/jpm14030247

**Published:** 2024-02-25

**Authors:** Seda Sarihan, Tolga Koyuncu, Leyla Kazancioglu, Sule Batcik, Hizir Kazdal

**Affiliations:** 1 Anesthesiology and Reanimation Clinic, Kaçkar State Hospital, 53300 Rize, Turkey; seda.kose@erdogan.edu.tr; 2 Department of Anesthesiology and Reanimation, Faculty of Medicine, Recep Tayyip Erdogan University, 53020 Rize, Turkeyhizir.kazdal@erdogan.edu.tr (H.K.)

**Keywords:** pleth variability index, general anesthesia, spinal anesthesia, perfusion index, prone position, lumbar disc hernia surgery

## Abstract

The study aimed to assess the pleth variability index (PVI) in patients undergoing lumbar disc herniation surgery under general and spinal anesthesia, exploring its correlation with fluid responsiveness, position, and hemodynamic parameters. Methods: This prospective study included 88 ASA 1–2 patients, aged 18–65, undergoing 1–3 h elective lumbar disc herniation surgery. Patients in groups GA and SA were observed for demographic, operative, and hemodynamic parameters at specified time points. (3) Results: PVI values were comparable between the GA and SA groups. After 250 mL of fluid loading, both groups showed a significant decrease in basal PVI at T2. Prone positions in GA exhibited higher PI values than in SA. The transition from a prone to supine position maintained PVI, while pulse and MAP decreased.; (4) Conclusions: PVI values were comparable in elective lumbar disc herniation surgery with general and spinal anesthesia. Both groups exhibited significant a PVI decrease at T2 after 250 mL of fluid loading, indicating fluid responsiveness. In general anesthesia, the prone position showed a lower MAP and higher PI values compared to spinal anesthesia. PVI and PI, sensitive to general anesthesia changes, could have beneficial additions to standard hemodynamic monitoring in spinal anesthesia management.

## 1. Introduction

According to the study conducted by Sean S. Rajaee and colleagues, the comparison of increasing rates of various surgical procedures, including lumbar disc herniation surgeries, between 1998 and 2008, demonstrated that spinal surgeries were performed at higher rates over time compared to other surgical procedures [1]. Lumbar disc herniation (LDH) surgery was performed via a posterior approach in the prone position. Generally, excessive bleeding or significant hemodynamic changes are not expected in this surgery. However, the difficulty of the surgical procedure and the comorbid conditions of the patients may affect both the anesthetic technique and the hemodynamic changes observed in the intraoperative period.

Depending on the expected duration of the surgical procedure and the patient’s comorbidities, both general and neuraxial anesthesia could be applied. These anesthetic methods have different effects on the patient’s hemodynamics in different periods of surgery. Depending on these differences and comorbid conditions, routine monitoring methods may be inadequate. In these cases, invasive arterial blood pressure monitoring, cardiac output monitoring, and similar methods can be used. These methods aim for healthy perioperative management by making mathematical inferences about the patient’s fluid requirement and cardiac output. However, more noninvasive methods are needed because these methods are invasive procedures that may cause various complications and are expensive.

In recent years, hemodynamic monitoring has been performed with a perfusion index (PI) obtained from the wavelength with a pulse oximetry device and pleth variability index (PVI), which is the measure of dynamic changes in PI during respiratory cycles, which provides non-invasive, continuous monitoring, can be easily applied and interpreted independently from the user. PVI monitoring provides a low risk of complications, reduced cost, and ease of use compared to other methods. The fluid management of PVI during surgical interventions [2] and its use for monitoring is steadily increasing [3].

Optimizing the intravascular volume status is very important to prevent postoperative organ failure and reduce complications [4]. When hemodynamic changes that may occur during anesthesia and surgery can be predicted in advance, unwanted hemodynamic effects are prevented.

Several studies have demonstrated the efficacy of PVI for targeted fluid therapy under general anesthesia [3,5]. In recent years, studies with patients breathing spontaneously during spinal anesthesia have been added to the literature [6,7]. But this is not a sufficient number.

Regarding PVI, a new noninvasive hemodynamic monitoring method, we evaluate hemodynamic monitoring and fluid response in these cases with PVI in addition to standard monitoring since there are no studies in the literature on spontaneous breathing in lumbar disc herniations. When reviewing the literature, we wanted to make an original contribution to the studies mostly performed under general anesthesia with case examples in which we can compare both anesthesia methods.

The aim of this prospective observational study is to evaluate the relationship of PVI values with position and other hemodynamic monitor parameters in patients under general anesthesia and spinal anesthesia for LDH operations.

## 2. Materials and Methods

A prospective observational study was planned with the approval of the Recep Tayyip Erdoğan University Non-Interventional Clinical Research Ethics Committee with the decision number 2021/122 dated 24 June 2021. Patients who wanted to be included in our study were informed in detail about the aims of this study and gave a voluntary consent form. This study was conducted in accordance with the Declaration of Helsinki, revised in 2013.

Patients in the ASA I-II risk group, aged 18–65 years, scheduled for elective lumbar disc herniation surgery under spinal or general anesthesia were included in the study. Exclusion criteria were emergency cases, pregnant women, children under 18 years of age and adults over 65 years of age, a body temperature < 36 °C, patients with peripheral arterial disease, valvular heart disease, arrhythmia, severe respiratory disease (advanced COPD, asthma), patients who underwent spinal anesthesia and then underwent general anesthesia due to inadequate analgesia, chronic renal disease, and patients with more than two levels of disc herniation planned and who were expected to have a long operation time were excluded from the study because they would disrupt the correlation between subjects. If signs of nerve damage were detected during the patients’ preoperative examinations or if the patients preferred general anesthesia, specifically those who were anxious and did not desire spinal anesthesia, these patients were included in the general anesthesia group, considering the potential impact of stress on PVI. Other patients were randomly divided into the following two groups: one receiving general anesthesia and the other receiving spinal anesthesia. General anesthesia; Group GA, and spinal anesthesia; Group SA. All patients were anesthetized by anesthesiologists with similar levels of experience.

Demographic data such as age, height, body weight, gender, and operative data (the duration of anesthesia, duration of operation, total intravenous (iv) fluid, and total amount of bleeding) were recorded. Among the routine hemodynamic monitoring values, pulse rate, mean arterial pressure (MAP), SpO_2_, respiratory rate, end-tidal CO_2_, tympanic temperature values, and PI and PVI values were recorded with a Massimo Mighty Sat Rx fingertip pulse oximetry device. PVI measurements were performed on all patients as a standard procedure, using the arm without administering intravenous fluids or conducting noninvasive blood pressure (NIBP) measurements. After placing the patients in the prone position, all factors that could disrupt finger perfusion were assessed, and the positioning of the patient’s arms were adjusted accordingly. Longitudinal pillows were used for all patients to ensure there was no change in intrathoracic and intra-abdominal pressure. These hemodynamic values were recorded intraoperatively and postoperatively at the following intervals: baseline values recorded in the supine position without preoperative sedation and IV fluid administration were recorded as T0. Sedation with 0.02 mg/kg of IV midazolam was applied, and 250 mL of IV crystalloid was administered over 10 min, and the values measured in the supine position were recorded as T1. As a perioperative routine, 3 mL/kg/h of crystalloid solution was planned as maintenance fluid therapy for all patients. General anesthesia was induced with 2 mg/kg of propofol, fentanyl at 2 mcg/kg, and rocuronium at 0.6 mg/kg via IV. Endotracheal intubation was performed with a spiral intubation tube 2 min after induction. Sevoflurane (2%) was used for inhalation anesthesia, remifentanil at 0.1–0.2 mcg/kg/min was used for IV anesthesia, and a mixture of air (60%) and oxygen (40%) was used for oxygenation. Ventilation was performed in the volume-controlled mode with a tidal volume of 6–8 mL/kg (according to ideal weight), a PEEP of 5 cm of H_2_O, and a respiratory rate with end-tidal CO_2_ between 32 and 35 mmHg. Spinal anesthesia was performed in the sitting position. After the local antisepsis conditions were met, hyperbaric bupivacaine at 0.5% and 15–17.5 mg of hyperbaric bupivacaine at 0.5% were injected into the subarachnoid space with a 25G Quincke-tipped spinal needle, avoiding the L3–L4 or L4–L5 space with lumbar disc herniation. Patients who were hospitalized after a spinal block were followed in the supine position for 10 min. Patients with sensory and motor block were placed in the prone position using long cylinder pillows for the operation. T2 was recorded at 15 min, T3 at 30 min, T4 at 45 min, and T5 at 60 min in the prone position. The patient was placed in the supine position after the surgical procedure and the measurement time in the follow-up in the post-anesthesia care unit was recorded as T6. 

When the systolic blood pressure fell below 90 mmHg after induction or during the continuation of surgery, 5 mg of ephedrine (total 30 mg) was administered, and 250 mL of IV bolus crystalloid fluid replacement was performed in cases with persistent hypotension despite this treatment. Intraoperative and postoperative complications were recorded.

### Statistical Analysis

Based on the percentage measurement values of the methods to be studied in the literature review, the total sample size was found as *n* = 78 using the G-POWER program with an effect size of 0.65, a power of 80%, and a margin of error of 0.05.

Statistical analyses were performed using the SPSS 11.5 for Windows, (Chicago, IL, USA) package program. In addition to descriptive statistical methods (mean, standard deviation, median, frequency, ratio, minimum, maximum), the distribution of the data was evaluated using the Shapiro–Wilk test. In the evaluations, intergroup comparisons of quantitative variables with a normal distribution were evaluated using an independent sample *t*-test, comparisons of which without normal distribution were evaluated using the Mann–Whitney U test, and comparisons of qualitative variables were evaluated using the Chi-square test and Fisher’s exact test (in case of expected value < 5). A dependent samples *t*-test was used to investigate time-dependent changes in quantitative data.

## 3. Results

The mean age of the patients was 49.5 ± 11 years, and the mean BMI (body mass index) was 28.5 ± 5 kg/m^2^. The mean duration of anesthesia was 94 ± 25 min (min), and the mean duration of operation was 80 ± 26 min. The demographic and operative data of the patients are given in Table 1.

In intergroup comparisons, age, weight, height, BMI, duration of anesthesia, operative time, amount of bleeding, chronic diseases, and ephedrine use were similar in both groups (*p* > 0.05).

The comparison of hemodynamic variables between groups at each time point is given in Table 2. According to this, pulse rate and PWV were found to be statistically significantly lower in Group GA compared to Group SA at T3, T4, and T5 in the pronated position (T3; *p* = 0.024, T4 and T5; *p* = 0.001, *p* < 0.05) (Table 2).

Time-dependent changes in PI and PVI were compared between the groups (Table 3). The measured PVI values were similar in group GA and group SA (*p* > 0.05). When we looked at the PI values, it was seen that the values in the T2, T3, T4, and T5 prone positions were statistically significantly higher in group GA compared to group SA (*p* = 0.01, *p* < 0.05).

**Figure 1:**
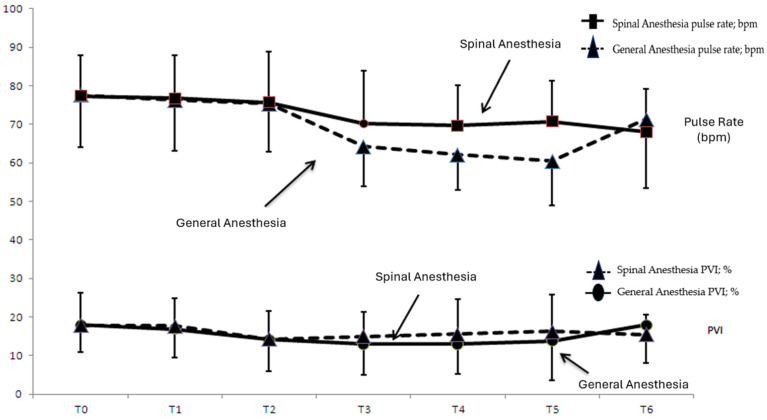
Time-dependent changein pulse rate(rpm/min) and PVI(%) values in group SA and group GA. X axis: T0; supine 0 min, T1; supine 10 min, T2; prone 15 min, T3; prone 30 min, T4; prone 45 min, T5; prone 60 min, T6; supine post-op. Y axis: The unit of measurement for the pulse rate values in the figure is rpm/min. The unit of measurement for the PVI values represents a percentage (%).

**Figure 2:**
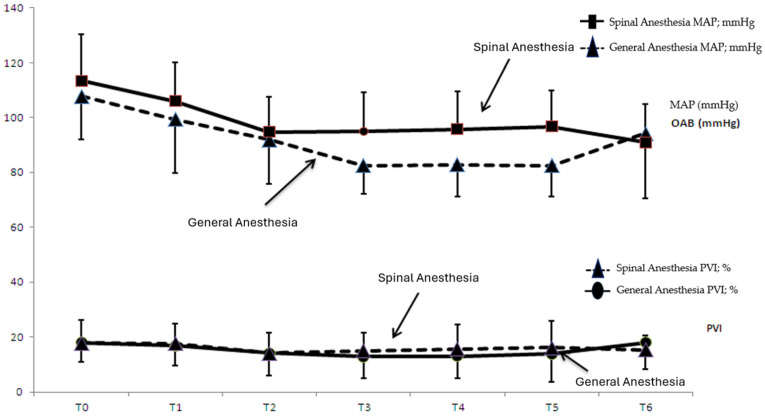
Time-dependent change in MAP and PVI values in group SA and group GA. X axis T0; T0; supine 0 min, T1; supine 10 min, T2; prone 15 min, T3; prone 30 min, T4; prone 45 min, T5; Prone 60 min, T6; supine post-op. Y axis: The unit of measurement for the MAP values in the figure is mmHg. The unit of measurement for the PVI values represents a percentage (%).

Figure 1 shows the time-dependent change in pulse rate and PVI values in group SA and group GA, while Figure 2 shows the time-dependent change in MAP and PVI values in group SA and group GA.

When the consecutive time measurements of the transition from the supine to the prone position were analyzed, the decrease in PVI values at T2, T3, T4, and T5 in group GA compared to T0 was statistically significant (T2; *p* = 0.001, T3; *p* = 0.001, T4; *p* = 0.004, T5; *p* = 0.044; *p* < 0.05).

In group GA, there was a statistically significant decrease in the PVI value at supine T1 compared to prone T3 (T1–T3; *p* = 0.012, *p* < 0.05). In group SA, there was a statistically significant decrease in supine T1 PVI values compared to prone T2 (*p* = 0.044, *p* < 0.05).

MAP values were compared according to measurement times (Table 4); a statistically significant decrease was observed in T0 MAP values at all times in both groups. From T1 to T2, a statistically significant decrease in MAP was observed in both groups. When we looked at the T1–T3 comparison, the decrease in MAP was significant in both groups (*p* = 0.001, *p* < 0.05). When we looked at the comparison of T2–T3 times, a statistically significant decrease was observed only in group GA. In the transition from the prone position to the supine position, a significant increase was observed in group GA (*p* = 0.011, *p* < 0.05), while a significant decrease was observed in group SA (*p* = 0.006, *p* < 0.05).

## 4. Discussion

In our study, we planned an observational study by adding noninvasive, easy-to-apply PVI monitoring to the perioperative follow-up parameters. 

There are many studies showing the efficacy of PVI in targeted fluid therapy under general anesthesia [3,5]. However, studies in spontaneously breathing patients are limited in the literature. During hemodialysis with ultrafiltration, a study indicated an increase in PVI values, demonstrating that this information could provide clinicians with useful insights into monitoring the volume status of patients with spontaneous breathing [8]. Additionally, studies evaluating the intravascular volume status of PVI during the Transurethral Resection of the Prostate (TURP) have been encountered [9]. 

There are no studies comparing PVI values in the same type of surgery and with two different anesthesia methods. When we statistically analyzed standard hemodynamic parameters and the PVI values of patients undergoing LDH surgery under general and spinal anesthesia, we discovered that PVI levels were similar in both groups. 

In the statistical analysis of the standard hemodynamic parameters of both groups, pulse and MAP values at T3, T4, and T5 were statistically significantly lower in group GA compared to group SA. The fact that the PVI value was correlated with static hemodynamic parameters in some time periods contrary to expectations may be related to the unreliability of the standard monitoring parameters we use in perioperative hemodynamic evaluations. There were cases where hemodynamic monitoring was not adequate due to the activation of compensatory mechanisms according to the changes in the hemodynamics of the patients or due to the drugs used age, and anesthesia method affecting the compensatory mechanisms. 

During LDH surgeries, various materials are used for positioning the patient in the pronated position, and depending on the type of materials used, intra-abdominal and intra-thoracic pressure increases may alter heart–lung interactions during mechanical ventilation. In a prospective study conducted by Çevikkalp et al. on 100 patients aged 18 and above undergoing elective posterior lumbar stabilization surgery with ASA I-II classification, they compared the classic calculation method and CVP monitoring for intraoperative fluid management with fluid loading based on PVI changes. This study indicates that the noninvasive nature of the PVI method and its ability to provide more accurate results in assessing the patient’s intravascular volume demonstrate the value of PVI as a valuable method during surgery [4]. When we review studies evaluating fluid responsiveness in patients in the prone position in the literature, Yang et al. found that SVV and PPV values showed fluid responsiveness at lower threshold values in supine compared to patients who were placed in the prone position using a Wilson frame [10]. Biais et al. discovered that when people were put in the prone position with four-pad support, the threshold values for pulse pressure variation (PPV) rose in the pronated posture [11]. In the study by Kim et al., in which 58 patients who were due to undergo lumbar stabilization were prone using a Jackson table, fluid loading was performed in both the supine and prone positions. The effect on PVI and PPV was evaluated by changes in CI and SVI. The PPV threshold value was found to be lower in the prone position than in the supine position, and the threshold value for PVI was found to be 8% in both positions. Poon et al. investigated the effect of the prone position on hemodynamics during lumbar spinal surgery and showed that the prone position did not cause any change in SVB but caused a decrease in SV and CI [12]. When examining these studies, the results may vary according to different criteria determined by the researchers, such as the use of different materials for the prone position and the selection of different fluidloading methods. All patients in our study were placed in the prone position using long cylinder pillows, which have less effect on cardiac performance. When the effect of hemodynamic measurements in the prone position was evaluated, the PVI values at T2–T3–T4–T5 time points in the prone position in the general anesthesia group were significantly lower than the baseline T0 values. In the spinal anesthesia group, only the PVI values at the T2 time point were lower than the baseline T0. When we looked at the MAP values at T0 and T1, a statistically significant decrease was observed at T2 and T3. While an increase in PVI values was expected due to the decrease in MAP caused by sympathetic block and a decrease in the vasomotor tone caused by the effect of spinal and general anesthesia, we evaluated the statistically significant decrease in PVI values compared to the baseline as fluid responsiveness.

We reviewed studies in the literature to evaluate the effect of tidal volume and spontaneous breathing on the PVI values of fluid responsiveness. The effectiveness of dynamic monitoring methods was emphasized to be effective in TVs above 8 ml/kg. However, we looked at a systematic review and meta-analysis investigating the fluid response dynamics of patients with a tidal volume (TV) < 8 mL/kg and TV > 8mL/kg. In this study, a total of 33 studies, including 1352 patients, were analyzed. PPV and SVV, which are fluid response predictors, were evaluated, and it was concluded that they reliably predict fluid response in TVs less than 8 mL/kg [1]. In our study, we adjusted the TV value of patients in group GA to 6–8 mL/kg. Studies evaluating the efficacy of PVI in patients with spontaneous respiration were reviewed. Delerme showed that the PVI value decreased significantly after passive leg raising in spontaneously breathing patients, and they concluded that PVI may be valuable in evaluating fluid response [13]. Tomo and colleagues showed that the PVI value decreased significantly after passive leg raising in spontaneously breathing patients, and they concluded that PVI could detect early hemodynamic changes after acute blood loss [14].

Studies conducted with volunteers who underwent spinal anesthesia or who were spontaneously breathing were reviewed in the literature. In the study conducted by Öksüz et al. in 2019, they found that the PVI value measured immediately after spinal anesthesia was predictive of hypotension during a cesarean section, but the predictive power was weak. In the study, the PVI threshold value was found to be 18.5% [15]. In 2008, Keller et al. performed a passive leg lift test in spontaneously breathing volunteer patients and evaluated PVI fluid response according to CO increase. They reported that a PVI value above 19% was predictive of fluid response, albeit weakly [13]. Kuwata et al. found that a PVI value >18% measured before spinal anesthesia in 50 patients undergoing a cesarean section predicted hypotension [7]. Sun et al. found that PVI values measured before a cesarean section in 85 pregnant women were significantly higher in patients who developed intraoperative hypotension compared to those who did not [16]. We also examine the study conducted by Küpeli et al. in 2020; geriatric patients who underwent spinal anesthesia were evaluated, and it was observed that the PVI values measured preoperatively in this group who developed hypotension were higher than those who did not develop hypotension, but this was not statistically significant [17]. In our study, PVI values in the spinal anesthesia group and the general anesthesia group were similar at all measurement times. We thought that PVI could be an effective predictive parameter in monitoring fluid responsiveness in patients undergoing spinal anesthesia, similar to patients undergoing general anesthesia.

Upon reviewing studies in the literature, when assessing the PVI threshold value in relation to changes in SVV, CI, and SVI values for appraising fluid responsiveness in mechanically ventilated patients, no clear numerical value was found, and different values between 8% and 15% were found to be significant [6,18,19]. In a 2008 study by Cannesson et al. evaluating 25 patients undergoing coronary artery bypass grafting, the fluid loading response was evaluated according to an increase in the cardiac index and the PVI threshold value, which predicts a positive response to fluid response with 81% sensitivity, was found to be 14% [3]. 

When we look at a study conducted by Hummler et al. on rabbits, they observed a higher PI in the presence of an increased pulsatile portion in pulse oximetry due to a decrease in the vascular tone occurring under general anesthesia [20]. In our study, only the PI values in the prone position in group GA were significantly higher than the prone PI values in group SA. This could be related to the effect of general anesthesia on the vasomotor tone and the contribution of position to the preload. In group SA, PI values in the prone position were statistically significantly lower than supine PI values. Since the physiology of the pulse oximetric waveform and impaired peripheral blood flow due to various variables affecting it affect PI measurements, PVI values should be interpreted carefully considering these factors [21]. Assuming that the increase in PI values, which is thought to be related to the vasodilator effect of general anesthesia and the effect of the prone position on hemodynamics, and an indicator of an increase in cardiac output, we interpreted it as providing adequate perfusion in the organs even though there were lower MAP values compared to group SA. When we reviewed the intergroup statistical comparison of PI values in both groups according to the PVI threshold value, PI values measured under general anesthesia and in the prone position were higher than those measured in spinal anesthesia patients and were statistically significant. In intragroup evaluations, PI values were statistically similar. 

In a study by Broch et al. [21], who investigated the effect of different PI values on the accuracy of PVI to predict fluid responsiveness in patients undergoing elective coronary artery surgery via performing a passive leg elevation test, it was observed that the accuracy of PVI to predict fluid responsiveness was weakened when the perfusion status of the finger to which it was attached was not taken into account, and when PI > 4%, PVI significantly affected the ability to discriminate between fluid responders and non-responders. PVI has been shown to reliably predict fluid responsiveness in high perfusion states [21].

Therefore, since there may be many factors affecting PVI in the intraoperative process, it is recommended to evaluate PVI together with the PI value. PVI is thought to be a guide for targeted fluid therapy with the information it provides about the preload in hemodynamic monitoring.

### Limitations of this Study

First, only ASA 1–2 patients participated in this study. Therefore, the findings cannot be generalized to patients in the geriatric age group and patients with decompensated diseases. As with other dynamic indicators, its use in patients with cardiac arrhythmias is not recommended.

Since we did not evaluate systemic vascular resistance in our study, our results cannot be extended to patients receiving vasoactive drugs (adrenaline, dopamine, etc.). We did not use any other control dynamic monitoring method to compare the volume status with PVI.

## 5. Conclusions

PVI values were similar at all times in patients who underwent elective LDH and underwent general and spinal anesthesia. In the general anesthesia group, MAP values in the pronated position were lower than in group SA. PI values were significantly higher in group GA compared to group SA. PVI and PI, which were found to be sensitive to changes, as in general anesthesia, may be recommended for use in addition to standard hemodynamic monitoring in spinal anesthesia management. 

Static hemodynamic monitoring values do not give any findings in the early period due to the compensatory mechanisms that may develop. We believe that new monitoring methods that can provide rapid and continuous information about blood flow and cardiac output should be included in our general and spinal anesthesia practices. Therefore, we recommend the use of PVI, a noninvasive method, in addition to classical hemodynamic markers in perioperative individualized targeted fluid therapy. 

Since PVI is not included in routine hemodynamic monitoring, more large-scale studies are needed regarding its use in targeted fluid therapy for patients in whom significant perioperative hemodynamic changes are expected. Studies demonstrating the efficacy of PVI in spontaneously breathing patients with advanced hemodynamic monitors and the determination of PVI threshold values may provide a noninvasive method that can provide a rapid and continuous insight into blood flow and cardiac output in patients who do not require further monitoring.

This study paves the way for studies in which PVI efficacy is demonstrated in spontaneously breathing patients with advanced hemodynamic monitors. We believe that this noninvasive method, which can determine PVI threshold values in spontaneously breathing patients and provide a rapid and continuous idea about blood flow and cardiac output in patients without the need for further monitoring, can be included in standard monitors.

## Figures and Tables

**Table 1:** Demographic and operative characteristics of the patients (mean ± standard deviation, *p* < 0.05).

	Group GA (*n* = 46)	Group SA (*n* = 42)	*p*
Age (year)	48 ± 13	50 ± 10	0.509 ^Ω^
Sex F/M	19/27	21/21	0.413 ^£^
BMI (kg/m^2^)	27.5 ± 4	29 ± 5	0.059 ^Ω^
Anesthesia Duration (min)	97 ± 24	90 ± 26	0.174 ^Ω^
Operation duration (min)	82 ± 24	77 ± 28	0.354 ^Ω^
Amount of bleeding(ml)	84 ± 97	72 ± 71	0.620 ^Ω^
Ephedrine presence	5(%10.9)	6(%14.3)	0.628 ^£^
Tympanic temperature T1	36.6 ± 0.5	36.8 ± 0.4	0.263 ^Ω^
Tympanic heat postop	36.1 ± 0.2	36.3 ± 0.4	0.359 ^Ω^
Chronic diseases			
Hypertension	10 (%21.7)	10 (%23.8)	0.817 ^£^
Diabetes mellitus	8 (%17.4)	7 (%16.7)	0.928 ^£^
Coronary artery disease	0	2 (%4.8)	0.225 ^µ^
Asthma	1 (%2.2)	2 (%4.8)	0.466

^Ω^: independent sample *t*-test, ^£^: Chi-square test, ^µ^: Fisher’s exact test, BMI: body mass index kg/m^2^, min: minute, ml: milliliter, Group SA; spinal anesthesia, Group GA; general anesthesia.

**Table 2:** Time-dependent hemodynamic changes in patients within groups and comparison of hemodynamic changes between groups (mean ± standard deviation, *p* < 0.05).

	Group GA (*n* = 46)	Group SA (*n* = 42)	*p*Ω
Pulse rate (beats/min)			
T0	77 ± 13	77 ± 10	0.909
T1	76 ± 13	76 ± 11	0.882
T2	75 ± 12	75 ± 13	0.900
T3	64 ± 10	70 ± 13	0.024
T4	62 ± 9	69 ± 10	0.001
T5	60 ± 11	70 ± 10	0.001
T6	71 ± 17	68 ± 11	0.311
MAP (mmHg)			
T0	107 ± 15	113 ± 17	0.114
T1	99 ± 19	105 ± 14	0.083
T2	91 ± 16	94 ± 12	0.357
T3	82 ± 10	95 ± 13	0.001
T4	82 ± 11	95 ± 13	0.001
T5	82 ± 11	96 ± 13	0.001
T6	94 ± 23	90 ± 14	0.435
SpO_2_			
T0	97 ± 1	97 ± 1	0.672
T1	97 ± 2	96 ± 1	0.135
T2	98 ± 1	98 ± 1	0.408
T3	98 ± 1	98 ± 1	0.787
T4	98 ± 1	98 ± 1	0.560
T5	98 ± 1	98 ± 1	0.147
T6	95 ± 1	97 ± 1	0.305

*p*Ω: independent samples *t*-test Group GA; general anesthesia group, Group SA; spinal anesthesia group, MAP; mean arterial pressure, SpO_2_: peripheral oxygen saturation.

**Table 3:** Results of statistical comparison of time-dependent PI, PVI changes within groups and PI, PVI changes between groups (mean ± standard deviation, *p* < 0.05).

	Group GA (*n* = 46)	Group SA (*n* = 42)	*p*Ω
PI			
T0	2.6 ± 2.4	3.7 ± 2.7	0.052
T1	3.6 ± 3.3	4.5 ± 3.2	0.188
T2	6.6 ± 9.3	2.0 ± 2.4	0.002
T3	5.8 ± 4.3	2.2 ± 3.7	0.001
T4	4.8 ± 3.4	1.4 ± 1.4	0.001
T5	4.7 ± 3.1	1.2 ± 1.2	0.001
T6	3.3 ± 5.2	2.0 ± 1.9	0.121
PVI			
T0	17.9 ± 7.1	17.7 ± 8.3	0.907
T1	16.8 ± 7.4	17.6 ± 7.0	0.619
T2	14.0 ± 8.2	14.2 ± 7.3	0.928
T3	12.8 ± 7.8	14.9 ± 6.4	0.195
T4	13.0 ± 7.9	15.5 ± 9.0	0.175
T5	13.7 ± 10.1	16.2 ± 9.5	0.308
T6	17.9 ± 9.8	15.3 ± 5.1	0.135
SpO_2_			
T0	97 ± 1	97 ± 1	0.672
T1	97 ± 2	96 ± 1	0.135
T2	98 ± 1	98 ± 1	0.408
T3	98 ± 1	98 ± 1	0.787
T4	98 ± 1	98 ± 1	0.560
T5	98 ± 1	98 ± 1	0.147
T6	95 ± 1	97 ± 1	0.305

Ω: independent samples *t*-test T0; supine 0 min, T1; supine 10 min, T2; prone 15 min, T3; prone 30 min, T4; prone 45 min, T5; prone 60 min, T6; supine post-op, group GA; general anesthesia group, group SA; spinal anesthesia group.

**Table 4:** Results of the statistical comparison of time-dependent PI, PVI changes within groups and PI, PVI changes between groups (mean ± standard deviation, *p* < 0.05).

MAP (mmHg)	Group GA (*n* = 46) *p*^β^	Group SA (*n* = 42) *p*^β^
T0–T1	0.006	0.001
T0–T2	0.001	0.001
T0–T3	0.001	0.001
T0–T4	0.001	0.001
T0–T5	0.001	0.001
T0–T6	0.001	0.001
T1–T2	0.044	0.001
T1–T3	0.001	0.001
T2–T3	0.001	0.839
T3–T4	0.933	0.181
T4–T5	0.455	0.236
T5–T6	0.011	0.006

Results of the intergroup comparison of time-dependent changes (one-way analysis of variance in repeated measures), ^β^: paired *t*-test. T0; supine 0 min, T1; supine 10 min, T2; prone 15 min, T3; prone 30 min, T4; prone 45 min, T5; prone 60 min, T6; supine postop, group GA; general anesthesia group, group SA; spinal anesthesia group.

## Data Availability

The data used in this study are available upon reasonable request.
